# Regulatory Interactions Between Neutrophils, Tumor Cells and T Cells

**DOI:** 10.3389/fimmu.2019.01690

**Published:** 2019-07-18

**Authors:** Hans-Heinrich Oberg, Daniela Wesch, Shirin Kalyan, Dieter Kabelitz

**Affiliations:** ^1^Institute of Immunology, Christian-Albrechts-University of Kiel, University Hospital Schleswig-Holstein, Kiel, Germany; ^2^Clinical Research Development Laboratory, Department of Medicine, BC Children's Hospital Research Institute, University of British Columbia, Vancouver, BC, Canada

**Keywords:** bispecific antibody, cytotoxicity, gamma/delta T cells, neutrophils, pancreatic ductal adenocarcinoma, T lymphocytes, tumor microenvironment, zoledronic acid

## Abstract

Apart from their activity in combating infections, neutrophils play an important role in regulating the tumor microenvironment. Neutrophils can directly kill (antibody-coated) cancer cells, and support other immune anti-tumoral strategies. On the other hand, neutrophils can also exert pro-tumorigenic activities *via* the production of factors which promote cancer growth, angiogenesis and metastasis formation. The balance of anti- and pro-cancer activity is influenced by the particularly delicate interplay that exists between neutrophils and T lymphocytes. In murine models, it has been reported that γδ T cells are a major source of IL-17 that drives the recruitment and pro-tumorigenic differentiation of neutrophils. This, however, contrasts with the well-studied anti-tumor activity of γδ T cells in experimental models and the anti-tumor activity of human γδ T cells. In this article, we first review the reciprocal interactions between neutrophils, tumor cells and T lymphocytes with a special focus on their interplay with γδ T cells, followed by the presentation of our own recent results. We have previously shown that zoledronic acid (ZOL)-activated neutrophils inhibit γδ T-cell proliferation due to the production of reactive oxygen species, arginase-1 and serine proteases. We now demonstrate that killing of ductal pancreatic adenocarcinoma (PDAC) cells by freshly isolated resting human γδ T cells was reduced in the presence of neutrophils and even more pronounced so after activation of neutrophils with ZOL. In contrast, direct T-cell receptor-dependent activation by γδ T cell-specific pyrophosphate antigens or by bispecific antibodies enhanced the cytotoxic activity and cytokine/granzyme B production of resting human γδ T cells, thereby overriding the suppression by ZOL-activated neutrophils. Additionally, the coculture of purified neutrophils with autologous short-term expanded γδ T cells enhanced rather than inhibited γδ T-cell cytotoxicity against PDAC cells. Purified neutrophils alone also exerted a small but reproducible lysis of PDAC cells which was further enhanced in the presence of γδ T cells. The latter set-up was associated with improved granzyme B and IFN-γ release which was further increased in the presence of ZOL. Our present results demonstrate that the presence of neutrophils can enhance the killing capacity of activated γδ T cells. We discuss these results in the broader context of regulatory interactions between neutrophils and T lymphocytes.

## Introduction

Polymorphonuclear neutrophils are bone marrow-derived white blood cells which account for 50 to 70% of leukocytes circulating in the peripheral blood. They are highly mobile and are generally considered to be short-lived but can increase their longevity upon activation during infection (1). Despite their characteristic morphology, neutrophils display an enormous functional plasticity which correlates to some extent with the expression of cell surface markers and the production of cytokines, chemokines, antimicrobial peptides (AMP) and bioactive molecules like serine proteases, arginase-1 and reactive oxygen species (ROS) (2). Neutrophils constitute perhaps the most important cellular component of innate immunity, playing an indispensable role in the immune defense against microbes like bacteria and fungi (3). They attack microbes through phagocytosis followed by degranulation or through the release of noxious substances, including granule-derived compounds like antimicrobial peptides, reactive oxygen species (ROS), and nitric oxide species (NOS), in addition to the extrusion of extracellular fibrillary networks termed neutrophil extracellular traps (NETs) (4). NETs are composed of nuclear material like DNA and histones and are decorated by proteins from neutrophil granula. Trapping of microorganisms and subsequent exposure to granule-derived proteins leads to their disposal, a process termed NETosis (4). Beyond their immediate role in innate immunity, it has become increasingly clear that neutrophils can also directly interact with other cells of the innate (e.g., Natural Killer [NK] cells and dendritic cells [DCs]) and adaptive (B cells, T cells) arms of the immune system. As such, neutrophils have been shown to produce, in a context dependent manner, a plethora of cytokines and chemokines (2, 5). Similar to other immune cells, the local micromilieu shapes the functional differentiation pathways of neutrophils. Thus, they can acquire a pro-inflammatory (type 1) phenotype associated with the production of cytokines such as IL-1, IL-6, TNF-α, or IL-17, or an anti-inflammatory (type 2) phenotype associated with production of cytokines like IL-1Rα (receptor agonist) and TGF-β (2, 4). It should be emphasized, however, that there is some controversy with respect to the reported expression/production of some factors (like IL-17) by human neutrophils. Important technical issues have to be taken into account (2, 6).

### Neutrophil Interactions With Tumor Cells

Neutrophils play important roles in cancer biology which may include both pro-tumorigenic and anti-tumorigenic activities, depending on the tumor type, the cellular microenvironment, and the constellation of immune modulating factors present. These multifaceted aspects have been summarized in several excellent recent review articles (7–10). Interestingly, increased numbers of neutrophils are frequently present in the peripheral blood of patients with various cancer types, correlating with less favorable prognosis (11). The phenotypic diversity and plasticity of circulating neutrophil subpopulations in cancer patients is reflected by physical properties such as density. Sagiv and coworkers identified three populations of neutrophils in the blood of cancer patients, consisting of low density large immature granulocyte-like myeloid-derived suppressor cells (G-MDSC) and mature neutrophils which exert pro-tumorigenic activity, and high density small mature neutrophils with anti-tumor activity (12). Conventional Ficoll-Hypaque density gradient centrifugation separates these neutrophil subsets as low density pro-tumorigenic neutrophils remain on top of the gradient together with monocytes and lymphocytes (i.e., the mononuclear fraction) while high density neutrophils with anti-tumor properties sediment to the bottom together with red blood cells (13). How can neutrophils mediate anti-tumor activity? It was noted already in the early 1980's that neutrophils can kill various tumor cell lines upon extended *in vitro* co-culture with tumor cells (14). More recently, it was observed that neutrophils from certain healthy donors were capable of killing several established human tumor cell lines but not primary epithelial cells; whereas neutrophils from lung cancer patients were much less active (15). Further analysis revealed that the activation of signaling pathways including PI3 kinase and p38 kinase increased the sensitivity of the selected tumor cells to neutrophil killing. In this study, cytotoxicity was determined by the Real-Time Cell Analyzer (RTCA) system which measures the decrease of impedance over time when adherent target cells detach from the bottom of culture wells as a consequence of lysis. Attempts to identify the mechanism of neutrophil killing of tumor cells in these studies pointed to a role of hydrogen peroxide (H_2_O_2_) since catalase significantly reduced the extent of tumor cell lysis (15). Recently, it was discovered that H_2_O_2_ secreted by neutrophils induces a lethal influx of Ca^2+^ in tumor cells which is mediated by the transient receptor potential cation channel, subfamily M, member 2 (TRPM2), a ubiquitously expressed H_2_O_2_-dependent Ca^2+^-permeable channel that is frequently upregulated in cancer (16). Interestingly, the expression of TRPM2 (and thus the sensitivity to neutrophil killing) is up-regulated during the epithelial-to-mesenchymal transition (EMT), rendering mesenchymal cells more susceptible to neutrophil cytotoxicity, while cells expressing lower levels of TRPM2, as observed during mesenchymal-to-epithelial transition (MET), are protected from neutrophil killing (17). In addition to the H_2_O_2_-dependent “spontaneous” cytotoxicity, neutrophils are potent mediators of Fc receptor-dependent antibody-dependent cellular cytotoxicity (ADCC) against antibody-opsonized tumor cells [discussed in (7)]. The antibody isotype plays an important role in triggering efficient ADCC. It appears that IgA antibodies targeting the FcαRI (CD89) expressed on neutrophils are most effective in this respect (9, 18). The mechanism of how neutrophils actually execute ADCC has been recently identified as trogoptosis; a process which involves intimate CD11b/CD18-dependent conjugate formation facilitating neutrophil antibody-opsonization leading to necrotic tumor cell death (19).

As briefly discussed, subsets of neutrophils can exert anti-tumor activity. However, a large body of evidence indicates that neutrophils actually promote tumorigenesis and metastasis formation through a plethora of mechanisms (6). This is supported by studies showing that the presence of tumor-associated neutrophils (TANs) correlates with a poor prognosis in different cancers (9, 10, 20–22), although this is not a generally valid observation (7, 23). An important aspect to consider when dissecting pro- vs. anti-tumorigenic neutrophilic functions is that results obtained from well-defined murine model systems may not always reflect the same role of neutrophils in corresponding human cancer diseases (24). Like macrophages, neutrophils can be categorized into type 1 and type 2 subsets. Type 1 neutrophils (N1) are pro-inflammatory and produce, amongst other factors, IL-12 and CCL3; whereas, N2 neutrophils are immunosuppressive and produce IL-10, CCL2 and high amounts of arginase (2). In the context of the tumor microenvironment, neutrophils are recruited and polarized into tumor-promoting N2 cells by tumor-derived factors, of which TGF-β has a major role (25). N2-polarized TANs possess a broad arsenal of effector mechanisms to support cancer growth, tumor metastasis and angiogenesis. These include (but are not limited to): the production of elastase, arginase-1, prostaglandin E_2_; the formation of NETs; and the secretion of pro-angiogenetic factors, like matrix metalloprotease 9 (MMP9) and VEGF (6, 9). There is also a role of neutrophils in promoting metastasis formation and tumor progression outside the primary tumor. A recent study reported that ovarian tumor-derived factors stimulated the neutrophil influx into the omentum and the local protrusion of NETs which were found to bind to ovarian cancer cells and thereby to promote metastasis to the omentum. NET formation is known to depend on peptidyl arginine deiminase 4 (PAD4) (26, 27). In their experimental model, Lee et al found that reduced NET formation, as observed in PAD4-deficient mice or following pharmacological inhibition of PAD4, was associated with reduced omental metastasis (28). Szczerba and coworkers recently described another pathway in which circulating neutrophils might contribute to metastasis formation (29). It was found that neutrophils in the peripheral blood can associate with rare circulating tumor cells. In comparison to the transcriptome of unassociated tumor cells, the tumor cells associated with neutrophils had up-regulated the expression of genes involved in cell cycle progression which lead to more efficient metastasis formation (29). Together, these new data broaden our understanding on the role of neutrophils in tumor metastasis formation and may develop new avenues for therapeutic intervention.

### Neutrophil Interactions With T Cells

T cells require two signals for activation, i.e., antigen recognition *via* the T-cell receptor (TCR) and a co-stimulatory signal typically provided by interaction between CD28 on T cells with corresponding B7 family members (CD80, CD86) on antigen-presenting cells (APC) (30). However, there are other co-stimulatory pathways such as ICOS/ICOS-ligand or CD40/CD40-ligand (CD154) which similarly play important co-stimulatory roles. The most potent APC are dendritic cells (DCs) which express high levels of MHC class II molecules (in addition to MHC class I), CD80 and CD86, and offer ample contact areas to T cells through their conspicuous dendrites. DCs process and present endogenous (e.g., viral) antigens to CD8 T cells *via* the MHC class I presentation pathway, but they also take up exogenous (e.g., bacterial) antigens and present those to CD4 T cells *via* the MHC class II presenting machinery. Notably, DCs can also “cross-present” exogenous antigens and bring them into the MHC class I pathway for presentation to CD8 T cells (31). While activated human T cells upregulate MHC class II molecules, and thereby can bind peptides and bacterial superantigens for presentation to other T cells, antigen processing and specifically cross-presentation is the domain of professional APCs, particularly DCs. T cell activation needs to be tightly controlled. To this end, T cells upregulate inhibitory receptors like CTLA-4 and PD-1, which upon interaction with their ligands CD80/86 and PD-1 ligand (PD-L1), respectively, deliver negative signals resulting in T cell growth arrest and exhaustion (32). Given that tumors as well as immunosuppressive cells in their microenvironment, like MDSC, frequently upregulate PD-L1 as a strategy to dampen efficient T-cell responses, the introduction of antibodies interfering with such pathways (“checkpoint inhibitors”) has been a major breakthrough in the treatment of certain cancers (33, 34). The use of checkpoint inhibitors may also interfere with the cancer-associated fibroblast (CAF)-induced PDL-1 expression on neutrophils, which functions to impair T cell-responses against hepatocellular carcinomas (35). In addition to the negative impact of PD-1 and CTLA-4 signaling, T cell activation is controlled by regulatory circuits involving FoxP3-positive regulatory T cells (Treg) and anti-inflammatory M2 macrophages, which are recruited by pro-tumorgenic neutrophils, and contribute to further suppressing cytotoxic T cell function (10). Multiple molecules have been implicated in Treg-mediated suppression, including CTLA-4, LAG3, TIGIT, IL-10, and ectoenzymes CD39 and CD73 expressed on Treg (36). Cells of the monocyte-macrophage lineage on the other hand are characterized by enormous plasticity. Pro-inflammatory M1 macrophages are induced under conditions of IFN-γ and TLR signaling, whereas IL-4 and IL-13 signaling skews polarization *via* STAT6 toward M2 macrophages (37). MDSC are yet another differentiation status of regulatory/suppressive myeloid cells. They comprise a heterogeneous group of cells where at least two groups (monocytic MDSC, granulocytic MDSC) can be differentiated on the basis of morphology and functional properties (38, 39). Overall, MDSC present in the tumor micromilieu contribute significantly to the immune escape in certain types of cancer by preventing efficient activation of tumor-infiltrating T cells. MDSC can inhibit IFN-γ production by T cells and degranulation of phosphoantigen-activated Vδ2 T cells (40, 41). Treatment of pancreatic ductal adenocarcinoma cells (PDAC)-patients with gemcitabine, the standard therapy for PDAC, can inhibit MDSC, while enhancing cross-presentation of tumor-associated antigens by DC (42). There is still a need to more fully characterize the influence of gemcitabine on the interaction of MDSC and γδ T cells. Treatment with other chemotherapeutic agents in combination with n-BP has shown to increase γδ T-cell cytotoxicity against tumor cells (43, 44).

In addition to MDSC, mesenchymal stromal cells (MSC) can enhance MDSC-mediated immunosuppression by inhibiting T-cell proliferation and IFN-γ production (45). MSCs have a broad functional repertoire and are crucial for tissue regeneration and homeostasis. As such, these stem cells are considered to hold significant therapeutic potential to reverse tissue damage in conditions with unrestrained neutrophil activation (46). Given their anti-inflammatory properties, MSCs are being investigated as a means to treat autoimmune diseases, graft vs. host disease (GvHD) and allograft rejection following transplantation (47). The property of MSCs to inhibit T cell proliferation is thought to act as a double-edged sword in the context of malignancy. One of the mechanisms driving this inhibition is the cytoplasmic tryptophan-catabolizing enzyme, indoleamine 2,3-dioxygenase (IDO), that is produced by human MSCs in response to inflammation and acts to deplete the essential amino acid tryptophan in the local environment, which results in the inhibition of the growth and survival of T cells (48–50). IDO is also produced by MDSCs and is considered as an important checkpoint molecule as it functions to enable cancer cells to subvert immune targeting (50, 51). Other immune suppressive mechanisms of MSCs involve their ability to program neutrophils into an immunosuppressive and tumor-promoting phenotype. CD11b^+^ Ly6G^+^ neutrophils isolated from bone marrow of normal mice or spleen of tumor-bearing mice inhibited T cell proliferation *in vitro* after coculture with TNF-α-primed MSC with and enhanced 4T1 tumor progression *in vivo*. These TNF-α-primed MSC conditioned neutrophils had upregulated arginase activity and the expression of iNOS, saa3, some cytokines and chemokines and their receptors. iNOS inhibition attenuated some of the suppressive effect of TNF-α-primed MSC pre-cocultured neutrophils on T cell proliferation (52).

How do neutrophils modulate T cell activation? Although classically considered as effector cells of innate immunity, it is obvious that neutrophils can exert both positive and negative effects on T cell activation. Under inflammatory conditions (e.g., *in vitro* culture with GM-CSF, IFN-γ, TNF-α, or *ex vivo* in patients with inflammatory diseases), neutrophils can acquire DC-like properties with upregulation of MHC class II and costimulatory molecules such as CD86 and CD83, thus being able to present antigen to T lymphocytes (53, 54). Moreover, neutrophils can support T-cell responses by secreting chemokines that are important for the recruitment of DCs or T cells, for example in the context of infection or contact hypersensitivity (55, 56). Depending on the specific cellular environment, neutrophils can thus positively modulate adaptive T-cell responses. It is clear, however, that neutrophils are armed with various strategies to effectively inhibit T-cell activation as well (9). Through production of ROS, suppressive granulocytic MDSC inhibit T-cell activation at the level of reduction of TCRζ expression, inhibition of NF-κB activation, as well as induction of apoptosis. The degranulation of serine proteases, such as elastase, proteinase-3, cathepsin-G, by primary granules inhibits T-cell activation by the inactivation of cytokines and their receptors. In addition, ariginase-1 released by tertiary granules cleaves the amino acid arginine, which is essential for T-cell activation. Furthermore, granulocytic MDSC also deplete the cellular environment of cystine through the ${\text{X}}_{\text{C}}^{-}$ transporter. In consequence, APC cannot reduce cystine into cysteine which is required for T-cell activation. Last but not least, neutrophils can upregulate PD-L1 and thereby deliver a negative signal to T cells *via* the PD-1 receptor (9, 35, 57). Approaches to investigate the modulation of T-cell activation *in vitro* using freshly isolated neutrophils showed that activated neutrophils can inhibit the polyclonal T-cell activation by CD3/CD28 antibodies, which was partially reversed by the ROS inhibitor catalase but not by NOS or myeloperoxidase (MPO) inhibitors. Suppression of T-cell activation by activated neutrophils was accompanied by significant, ROS-induced cell death (58). Other strategies which neutrophils use to inhibit T-cell activation include the production of suppressive cytokines, such as IL-10 and the upregulation of PD-L1. In a murine model of infection using *Mycobacterium bovis*, Doz et al. observed that neutrophils recruited by infected DCs produced a large amount of IL-10. It was further demonstrated in an OVA TCR transgenic model that IL-10 producing neutrophils specifically suppressed IL-17 but not IFN-γ production in OVA-specific T cells (59). In a different system, it was found that LPS-stimulated Treg induced IL-10 production in neutrophils in a cell contact-dependent manner. It was shown that LPS-activated Treg (but also exogenous IL-10) promoted specific histone modifications that activated the IL-10 genomic locus in neutrophils (60). Upregulation of PD-L1 on neutrophils as a means of T-cell suppression has been identified in various systems (9, 35). As an example, increased expression of PD-L1 was observed on neutrophils in the peripheral blood of patients infected with *Burkholderia pseudomallei*, and it was found that such neutrophils inhibited polyclonal T-cell activation in a PD-1/PD-L1 dependent manner (61). Furthermore, increased neutrophil expression of PD-L1 is also found in HIV-1 infected patients, again correlating with a PD-1/PD-L1 dependent inhibition of T-cell activation. In this study, IFN-α, the TLR7/8 agonist Resiquimod, and HIV-1 virions were identified as potent inducers of PD-L1 expression on neutrophils (62). Indeed, immune activation using TLR ligands or microbial products may be an effective therapeutic strategy to overcome cancer-associated immune suppression and increase the efficacy of anti-cancer cytotoxic T cell activity (63, 64).

### Neutrophil Interactions With γδ T Cells

γδ T cells comprise a small subset of CD3-positive T cells in the peripheral blood but account for a major population of intraepithelial lymphocytes in mucosal tissue such as the small intestine. The dominant population of γδ T cells in human peripheral blood expresses a TCR composed of the Vγ9 chain paired with Vδ2. With substantial interindividual variability, such Vγ9Vδ2 T cells (termed Vδ2 in the following sections) make up anywhere between 50 and 95% of peripheral blood γδ T cells in adult healthy donors (65). The TCR repertoire of intestinal γδ T cells is different; non-Vδ2 (i.e., Vδ1 or Vδ3) T cells co-expressing any of the available Vγ elements are predominant (66). γδ T cells play a major role in (local) immune surveillance as they sense stressed and transformed cells by their TCR and additional activating receptors like NKG2D (66, 67). In line with this, γδ T cells are potent cytotoxic cells and are known to kill a broad range of tumor cells in a MHC non-restricted but TCR and/or NKG2D-dependent manner (68, 69). The NKG2D receptor present on virtually all human γδ T cells (in addition to NK cells, CD8 T cells and a small subset of CD4 T cells) binds to corresponding ligands, such as MHC class I chain-related gene A or B (MICA/B) and members of the ULBP family expressed on tumor cells, thereby triggering PI3-kinase dependent signaling pathways leading to cytokine production and cytotoxic effector activity (70). The TCR of Vδ2 T cells recognizes pyrophosphate molecules which are intermediates of both the non-mevalonate and the dysregulated mevalonate pathways of isoprenoid synthesis in prokaryotic and eukaryotic cells, respectively. The prototypic microbial “phosphoantigen” (*E*)-4-Hydroxy-3-methyl-but-2-enyl pyrophosphate (HMBPP) exclusively activates human Vδ2 T cells at pico- to nanomolar concentrations (71, 72). The transient increase of γδ T cells in the peripheral blood during the acute phase of many bacterial and parasitic infections is due to the release of microbial phosphoantigens (73, 74). The intermediate isopentenyl pyrophosphate (IPP) of the eukaryotic mevalonate pathway of cholesterol synthesis is similarly recognized by Vδ2 T cells but requires much higher concentrations (in the micromolar range). While normal resting cells, including neutrophils (72), do not generate enough IPP to activate γδ T cells, many tumor cells have a dysregulated mevalonate pathway with increased IPP production and concomitant sensitivity to γδ T cell recognition and killing (75). The selective activation of human Vδ2 T cells by microbial or eukaryotic phosphoantigens requires the presence of the butyrophilin molecule 3A (BTN3A/CD277); in the absence of CD277, Vδ2 T cells are not activated by pyrophosphate molecules (76, 77). It appears that pyrophosphates bind to the intracellular B30.2 signaling domain of BTN3A1 resulting in a conformational change of the extracellular part of CD277, which is then recognized by the Vγ9Vδ2 TCR (78). Importantly, the intracellular production of IPP can be pharmacologically manipulated. Nitrogen-containing bisphosphonates such as zoledronic acid (ZOL) (which are in clinical use for the treatment of bone diseases) block an enzyme downstream of IPP synthesis in the cholesterol synthesis pathway, leading to the upstream accumulation of the γδ T cell-stimulating IPP (75). As a consequence, the uptake of nitrogen-bisphosphonates by monocytes but not by neutrophils within peripheral blood mononuclear cells (PBMC) induces a strong, selective expansion of Vδ2 T cells in the presence of recombinant IL-2 (rIL-2) (72, 79). Exposure of tumor cells to nitrogen-bisphosphonates drastically increases their sensitivity to γδ T cell-mediated killing (80). Transient activation and expansion of Vδ2 T cells is also observed *in vivo* upon application of ZOL and low-dose rIL-2 (81). Given that the abundance of γδ T cells among tumor-associated immune cells is a favorable prognostic marker (82) and in view of the developing strategies to apply γδ T cells for immunotherapy (83), it is important to consider the possible reciprocal interactions between γδ T cells and neutrophils (84). In mice, γδ T cells are an early source of IL-17 required for neutrophil migration in bacterial and fungal infections (85, 86). However, the same activity of murine γδ T cells might be detrimental in cancer as IL-17 producing γδ T cells were shown to promote metastasis formation in murine models of breast cancer, due to the mobilization of neutrophils which suppressed efficient CD8 T-cell responses (87). IL-17 producing γδ T cells can also recruit MDSC of monocytic and granulocytic origin, thereby again promoting tumor progression (88, 89). Overall, these results have raised the notion that γδ T cells play an ambiguous role in tumor immunity. While their potent cytotoxic activity against many cancers offers the promise for immunotherapy, the potential pro-tumorigenic activities of γδ T cells need to be targeted as well (90). In this context, it is of interest that tumor-associated neutrophils were recently shown to suppress IL-17 producing γδ T cells in the tumor microenvironment through the induction of oxidative stress (91). This finding corroborates previous data indicating that neutrophils can inhibit the *in vitro* activation of human γδ T cells (92). Here, the suppressive mechanism at play was deduced to be ROS production as it was abrogated in the presence of catalase (91). Using ZOL to activate and expand human Vδ2 T cells *in vitro* in the presence of exogenous rIL-2, we found that neutrophils inhibit γδ T-cell activation and proliferation. In addition to ROS, our results also pointed to a role of serine proteases and arginase-I in the γδ T-cell inhibition, based on the partial reversion by corresponding individual inhibitors and the complete reversion by the combination of inhibitors for ROS, serine proteases and arginase-1 (93). Even though neutrophils express CD277 and take up ZOL efficiently, they do not support Vδ2 T-cell activation, likely due to their strongly impaired production of IPP (72). Instead, they appear to suppress the activation of resting γδ T cells through the release of inhibitory molecules. Our observation that neutrophil serine proteases inhibit γδ T-cell activation (94) extends to their role in inhibiting conventional αβ T cells by membrane-associated proteinase 3 expressed by granulocytes (95).

In view of (i) the potent anti-tumor activity of γδ T cells, (ii) the complex interplay between neutrophils and tumor cells, and (iii) the reported reciprocal interactions between neutrophils and γδ T cells, we studied the modulation of anti-tumor cytotoxicity of short-term expanded human γδ T-cell lines by freshly isolated neutrophils and the effects of ZOL treatment.

## Results and Discussion

### Zoledronic Acid-Stimulated Neutrophils Diminish γδ T-Cell Cytotoxicity Against Pancreatic Ductal Adenocarcinoma Cells

**Figure 1:**
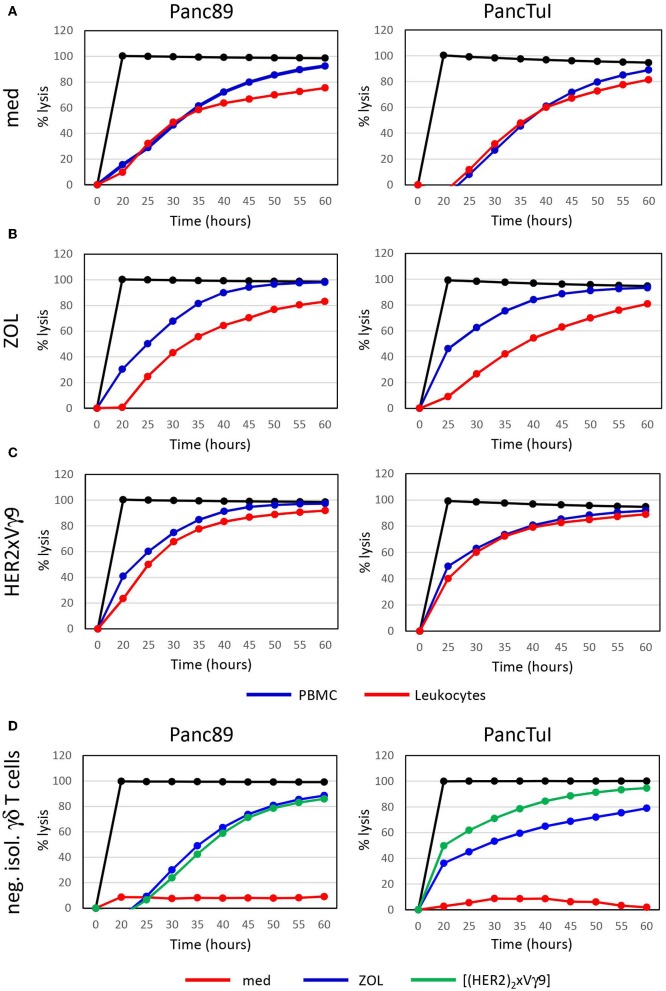
Zoledronic acid-stimulated leukocytes diminish γδ T cell-cytotoxicity against PDAC cells. **(A–C)** Cytotoxicity of 250 × 10^3^ PBMC (blue line) or 500 × 10^3^ leukocytes (red line), which each comprised ~7,500 γδ T cells or **(D)** 125,000 negatively isolated, resting γδ T cells (neg. isol. γδ T cells) in co-cultures with Panc89- (left panel) or PancTuI cells (right panel). Co-cultures were set up in rIL-2 medium only **(A)**, or were additionally stimulated with **(B)** zoledronic acid (ZOL, 2.5 μM) or **(C)** with bispecific Ab (bsAb) ([(HER2)_2_×Vγ9], 1 μg/mL). Cytotoxictiy was analyzed by RTCA. 5 × 10^3^ PDAC cells were seeded 24 h before addition of effector cells. Percentage of lysis (“% lysis”) was analyzed from RTCA data by calculating the normalized impedance of spontaneous lysis (cell growth of tumor cells in medium alone) in relation to the maximal lysis induced by 1% Triton-X-100 (black line) at indicated time points. Shown is one representative experiment with the same donor out of three independent experiments with different donors.

Zoledronic acid (ZOL) is an approved drug in clinical use for bone fragility disorders and cancer-associated bone disease. In addition to its role as an anti-resorptive agent, ZOL selectively activates human Vγ9Vδ2 T cells and induces their expansion when used in combination with rIL-2. A partial success of tumor reduction after application of ZOL together with rIL-2 was observed in several pilot studies, and this benefit can be further improved by combining adoptive transfer of activated γδ T cells together with ZOL and rIL-2 administration (68, 81, 96–101). ZOL is taken up *via* endocytosis by monocytes or tumor cells, a process which results in a strong selective expansion of γδ T cells and potentiation of their cytotoxic activity (75, 79, 80). Our previous reports demonstrated that neutrophils can also take up ZOL. This uptake, however, resulted in the release of neutrophil-derived hydrogen peroxide, serine proteases and arginase, which collectively inhibited proliferation and cytokine production of resting γδ T cells within purified leukocytes (93, 94). Here, we have performed additional studies to dissect the interaction between neutrophils and resting γδ T cells regarding their cytotoxicity against cancer cells. Using PDAC as target cells, we initially compared two experimental conditions: (i) Ficoll-Hypaque gradient separated peripheral blood mononuclear cells (PBMC) which contain resting γδ T cells and monocytes, but no neutrophils; (ii) red blood cell-lysed leukocytes, which contain all cells present in PBMC plus neutrophils. All co-cultures contained medium supplemented with rIL-2, and were additionally stimulated or not (Figure 1A) with ZOL (Figure 1B) or the bispecific antibody [(HER2)_2_xVγ9] (Figure 1C), both of which induce selective Vγ9Vδ2 γδ T-cell activation and cytotoxic effector functions. While γδ T cells within PBMC were able to exert their full cytotoxic activity against PDAC target cells with both ZOL or bispecific antibody treatments, γδ T cells within leukocytes showed an impaired cytotoxicity after activation with ZOL (Figure 1B) compared to the bispecific antibody (Figure 1C). These results suggest that the uptake of ZOL by neutrophils can inhibit cytotoxic γδ T-cell function, as previously shown for proliferative activation of γδ T cells (93). Whereas, the bispecific antibody, which specifically targets human epidermal growth receptor 2 (HER2)-expressing tumor cells and Vγ9-bearing γδ T cells, does not induce the same inhibitory activity of neutrophils (102–104). Since PBMC and leukocytes contain Natural Killer (NK) cells which could respond to rIL-2 alone (Figure 1A, medium control), we additionally applied negatively isolated resting γδ T cells in our studies. As shown in Figure 1D, negatively isolated, resting γδ T cells did not exert cytotoxic activity cultured in medium containing rIL-2. In contrast, the stimulation of the γδ T cells with ZOL or bispecific antibody drastically enhanced γδ T-cell cytotoxicity against PDAC cells in the absence of NK cells and other accessory cells (Figure 1D). Similar to the treatment with bispecific antibodies, stimulation with phosphoantigens that specifically activate γδ T cells, such as bromohydrin pyrophosphate (BrHPP), resulted in fully cytotoxic effector functions of γδ T cells in the presence of neutrophils (data not shown). In contrast to ZOL, phosphoantigens like BrHPP or HMBPP directly activate γδ T cells and do not appear to induce neutrophil burst or release of ROS and proteases that would inhibit γδ T cell-functions (105, 106). Recently, we reported that serine proteases released by neutrophils, such as proteinase 3, elastase and cathepsin G, decreased the cytotoxicity of freshly isolated, resting γδ T cells after their activation with BrHPP (94). The inhibition of IFN-γ and TNF-α production by resting γδ T cells in the presence of neutrophil-derived serine proteases (94) may play a key role in the reduced γδ T-cell cytotoxicity in the presence of neutrophils. ZOL, but not BrHPP, can trigger the release of ROS and serine proteases in neutrophils, which likely accounts for the observed differences in resting γδ T-cell activation within leukocytes in the presence of ZOL- vs. bispecific antibody- or BrHPP-stimulation (Figures 1B,C). To overcome ZOL-induced neutrophil-mediated suppression on the release of IFN-γ and TFN-α from resting γδ T cells, we cultured neutrophils with short-term activated γδ T cells, which are already continuously producing TNF-α and IFN-γ, and measured their cytotoxicity against PDAC cells with and without ZOL treatment.

Interestingly, clinical trials have shown that repetitive *in vivo* stimulation of γδ T cells with ZOL and rIL-2 can result in partially reduced tumor growth for a number of different types of cancers, including prostate cancer, advanced breast cancer and multiple myeloma. However, this repeated activation protocol was associated with exhaustion, anergy, and depletion of γδ T cells (81, 107, 108). This may be due to the simultaneous ZOL-induced release of serine proteases and ROS by neutrophils. In contrast, clinical trials using the adoptive transfer of γδ T cells that were short-term expanded with ZOL and rIL-2 more consistently reduced tumor growth in the context of advanced renal carcinoma, non-small-cell lung cancer, and other solid tumors (97, 100, 101), which suggests that pre-activation of γδ T cells can be helpful. Interestingly, the adoptive transfer of short-term expanded γδ T cells together with bispecific antibodies and rIL-2 reduced the growth of pancreatic tumors grafted into immunocompromised mice more significantly in comparison to adoptively transferred γδ T cells in conjunction with ZOL and rIL-2 (102).

Sun and colleagues demonstrated that IFN-γ and TNF-α, which are abundantly produced by NK cells, can convert tumor-promoting neutrophils into tumor-suppressing ones (109). The observation that an enhanced number of neutrophils in relation to lymphocytes has been associated with poor clinical outcome and reduced overall survival of cancer patients (110, 111) encouraged Sun and coworkers to target neutrophil effector functions as means of improving patient outcomes (109). Having shown that the presence of neutrophils apparently inhibits the γδ T-cell cytotoxicity stimulated by ZOL of resting γδ T cells (i.e., contained within leukocytes vs. PBMC), we therefore asked how neutrophils would impact on cytotoxic effector function of short-term expanded γδ T cells, which also produce high amounts of IFN-γ and TNF-α. To this end we analyzed whether the suppressive effect of neutrophils on γδ T cell-cytotoxicity could be overcome, similar to what has been observed with NK cells (109).

### Enhanced Anti-tumorigenic Effect of γδ T Cells Co-cultured With Autologous Neutrophils Against PDAC Cells

Neutrophils can act as a double-edged sword in cancer progression due to their remarkable heterogeneity and plasticity (9, 112). For instance, neutrophils can be polarized toward distinct phenotypes, not only by NK cells, but also by tumor derived signals (113). For example, Panc89 cells can produce high levels of IL-6 (unpublished observation), which is reported to induce N2 polarization of neutrophils (6, 114). IL-6, however, does not influence γδ T-cell cytotoxicity against tumor cells (115). In general, tumor-suppressing (N1 polarized neutrophils) are short-lived cells with mature phenotype and high cytotoxicity; whereas, tumor-promoting (N2 polarized neutrophils) are long-lived cells with immature phenotype and low cytotoxicity (6, 113).

**Figure 2:**
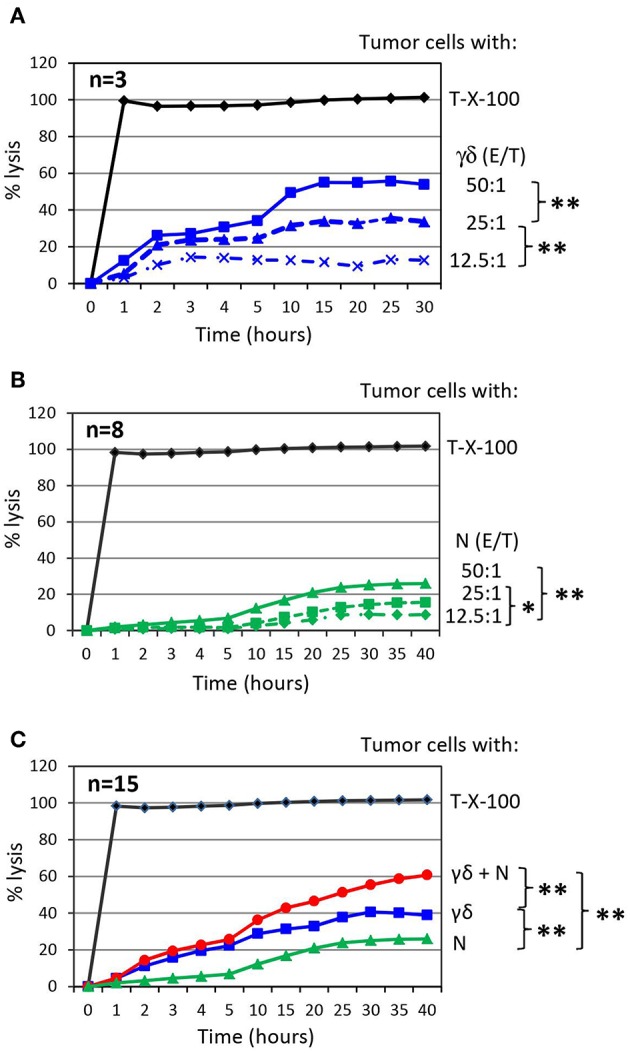
Co-culture of γδ T cells with autologous neutrophils in the presence of PDAC enhances cytotoxicity. **(A)** Cytotoxicity induced by short-term activated γδ T cells (γδ) at the indicated Effector/Target ratio (E/T). The mean of three individual samples for the indicated time points are shown. **(B)** Cytotoxicity induced by neutrophils (N) at the indicated Effector/Target ratio (E/T). E/T ratio was 12.5:1 (dashed line with x, 62.500 effector cells), 25:1 (dashed line with triangle, 125,000 effector cells) and 50:1 (solid line with square, 250,000 effector cells). The mean of eight individual samples for the indicated time points are presented. **(C)** Cytotoxicity of 250 x 10^3^ neutrophils (N, green line) or 125 × 10^3^ γδ T cells (γδ, blue line, E/T 25:1) alone or in combination (γδ + N, red line) against Panc89 cells was measured by RTCA. The mean of 15 individual samples for the indicated time points are shown. Percentage of lysis was analyzed from RTCA data by calculating the normalized impedance of spontaneous lysis (cell growth of tumor cells in medium alone) in relation to the maximal lysis induced by 1% Triton-X-100 (T-X-100, black line) at indicated time points. **(A–C)** Statistical analysis was performed by *t*-test. Significances are presented as *P*-Value; ^**^P < 0.01 and ^*^P < 0.05.

**Figure 3:**
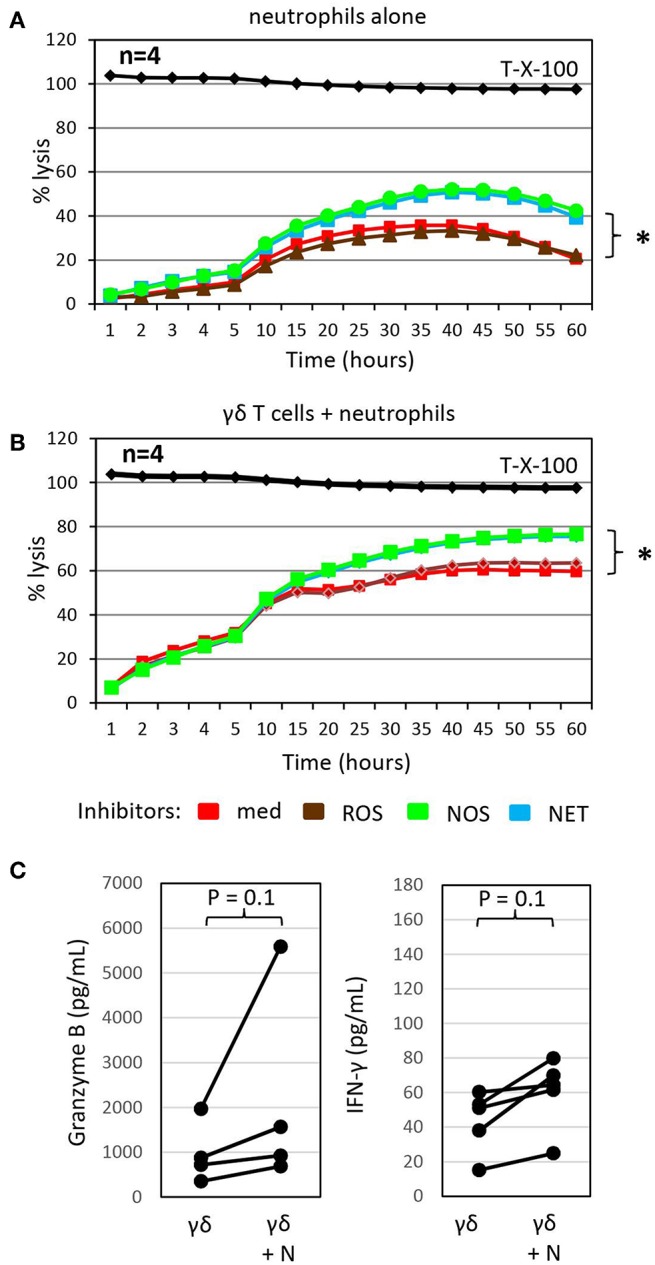
Inhibitors of NETosis and NO production slightly enhance γδ T-cell mediated cytotoxicity in the presence of autologous neutrophils and granzyme B and IFN-γ release. **(A)** Cytotoxicity of 250 × 10^3^ neutrophils alone or **(B)** in combination with 125 × 10^3^ γδ T cells (γδ + N, E/T 25:1) against Panc89 cells was determined by RTCA. Three hours before the addition of **(A)** medium or **(B)** γδ T cells, tumor cells and neutrophils were pre-incubated with inhibitors against radical oxide synthase (ROS, brown line; catalase, 4,500 U/mL), nitric oxid synthase (NOS, green line; N^G^,-N^G^-dimethyl-L-Arginine, 100 nM), NETosis (NET, blue line; GSK484, 10 μM) or no inhibitor (med, red line). The mean of four individual samples for the indicated time points are shown. Percentage lysis was analyzed from RTCA data by calculating the normalized impedance of spontaneous lysis (cell growth of tumor cells in medium alone) in relation to the maximal lysis induced by 1% Triton-X-100 (T-X-100, black line) at indicated time points. **(C)** In parallel, granzyme B and IFN-γ release was determined by ELISA from supernatants generated under the same conditions as the RTCA data and collected after 24 h. **(A–C)** Statistical analysis was performed by *t*-test. Significances are presented as *P*-Value; ^*^P < 0.05 or indicated significances.

To investigate the impact of purified neutrophils on the cytotoxic activity of short-term expanded γδ T cells against PDAC cells, we co-cultured target cells with γδ T-cells or neutrophils as effector cells with varying effector/target (E/T) ratios and analyzed cytotoxicity. We observed an E/T ratio-dependent lysis of PDAC cell line Panc89 by γδ T cells or neutrophils (Figures 2A,B). The highest E/T ratio showed very moderate lysis of Panc89 cells by neutrophils compared to that of γδ T cells with the same E/T ratio. More interestingly, when Panc89 cells were co-cultured with γδ T cells and neutrophils, lysis of tumor cells was significantly increased; up to 60 % in comparison to γδ T-cell effector cells alone with the 15 different blood donors tested (Figure 2C, red line). For the graphical presentation, we selected an E/T ratio at which both effector cells alone (γδ T cells: 25:1; neutrophils: 50:1) lysed the tumor cells to a similar extent. Although Panc89 cells were not completely lysed by either γδ T cells or neutrophils alone, a striking synergistic effect against Panc89 cells was observed. Lysis of Panc89 cells by γδ T cell occurs primarily through the release of granzymes and perforin (102, 116); whereas, neutrophils did not release granzymes, even after treatment with ZOL (unpublished observation). The inhibitory effects of neutrophils on tumor growth and tumor progression are mediated by different mechanisms (9, 117). After interaction with tumor cells, neutrophils can release ROS or NOS to trigger oxidative damage followed by cell death (15, 118), induce NETosis (117), or antibody-mediated trogocytosis (19). None of the direct cytotoxic mechanisms appear to play a major role for the observations made in our study since none of the applied inhibitors, which are described under section Synergism of neutrophils and γδ T cells toward PDAC cell lysis in more detail, inhibited the neutrophil-mediated cytotoxicity against PDAC cells (Figure 3A). Alternatively, activated neutrophils can release a wide array of cytokines, chemokines and proteases that influence the effector functions of other immune cells including T cells and NK cells (6, 23, 119, 120).

### Synergism of Neutrophils and γδ T Cells Toward PDAC Cell Lysis

To probe the potential indirect mechanism(s) by which neutrophils influence γδ T-cell cytotoxicity against tumor cells, we treated neutrophils co-cultured with Panc89 cells with different inhibitors of antimicrobial mediators that are known to be released by neutrophils for 3 h prior to the addition of γδ T cells. While catalase, an enzyme that degrades hydrogen peroxide, did not influence γδ T cell-mediated lysis of PDAC cells co-cultured with neutrophils, the NOS inhibitor, N^G^, N^G^ dimethyl L-arginine, and the PDA4 inhibitor, GSK484, which attenuates NETosis, both modestly enhanced lysis of Panc89 target cells (Figure 3B). Similar effects were seen with these inhibitors when neutrophils were cultured alone with Panc89 cells (Figure 3A). The observation that catalase did not influence PDAC lysis in these experiments suggests that neutrophil-derived superoxide anion (O2^**−**^) or hydrogen peroxide (H_2_O_2_) are not major contributors in this setting. O2^**−**^ can, however, also react with nitric oxide (NO) to form reactive nitrogen species (RNS), such as peroxynitrite (19). NO can be formed from arginine by the enzyme inducible nitric oxide synthase (iNOS) expressed by activated macrophages, which were not present in our cultures. The NOS inhibitor, N^G^, N^G^ dimethyl L-arginine, is an endogenous iNOS inhibitor, and it competes with endogenous L-arginine as a substrate for iNOS. L-arginine is an essential amino acid for T cells (19), and the addition of N^G^, N^G^ dimethyl L-arginine in our experiments showed some benefit to PDAC lysis. This suggests that L-arginine may be limiting for γδ T cells in our experimental set-up, and its addition could help support their anti-cancer effector functions. In addition, treatment with the NETosis inhibitor moderately enhanced γδ T cell-mediated lysis of PDAC cells, but did not reduce cytotoxicity of neutrophils (Figures 3A,B). This observation suggests that neutrophils may be producing some additional factor(s) (potentially cytokines or AMP) that indirectly influence granzyme B release from γδ T cells. In this context, it is of considerable interest that the release of granzyme B and IFN-γ by γδ T cells co-cultured with Panc89 cells was clearly enhanced in the presence of neutrophils compared to the cultures without neutrophils (Figure 3C). The increased release of cytotoxic mediators by γδ T cells can explain the enhanced cytotoxic activity against Panc89 cells (Figures 2C, 3C). Taken together, the results argue for a synergistic rather than an additive effect of γδ T cells and neutrophils in killing Panc89 cells. Riise and colleagues recently reported that the activation of neutrophils induced an increase in IFN-γ production in T cells (119). In line with these results, we observed that the presence of neutrophils served to potentiate γδ T-cell mediated tumor cytotoxicity, at least partly *via* enhanced degranulation and augmented Th1 cytokine release (Figures 2C, 3C). While IL-17 producing γδ T cells reportedly contribute to the expansion of granulocytic MDSC (89, 121), our study indicates that Th1-type γδ T cells do not induce immunosuppressive neutrophils.

### Zoledronic Acid Enhanced Cytotoxicity of Activated γδ T Cells Against PDAC Cells in the Presence of Neutrophils

**Figure 4:**
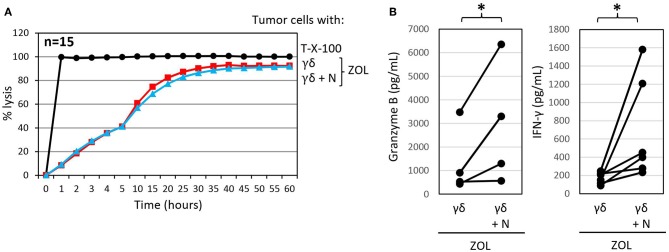
Zoledronic acid pre-treated autologous neutrophils do not influence γδ T-cell mediated cytotoxicity but further enhance granzyme B and IFN-γ release. **(A)** Cytotoxicity of 125 × 10^3^ γδ T cells alone or 250 × 10^3^ neutrophils in combination with 125 × 10^3^ γδ T cells (γδ + N, E/T 25:1, blue line) toward Panc89 cells was measured by RTCA. Tumor cells and neutrophils were pretreated with ZOL (2.5 μM) 3 h before addition of γδ T cells. Percentage lysis was analyzed from RTCA data by calculating the normalized impedance of spontaneous lysis (cell growth of tumor cells in medium alone) in relation to the maximal lysis induced by 1% Triton-X-100 (T-X-100, black line) at indicated time points. The mean of 15 individual samples for the indicated time points are shown. **(B)** Granzyme B and IFN-γ release was measured after 24 h by ELISA from supernatants generated under the same conditions as the RTCA experiments. The mean of four to six individual samples for the indicated time points are shown. Statistical analysis was performed by *t*-test. Significances are presented as *P*-Value; ^*^P < 0.05.

We previously reported that the PDAC cell line Pan89 cannot be completely lysed by γδ T cells unless they were additionally stimulated with selective γδ T cell agonists, such as ZOL or bispecific antibodies (69, 102). ZOL is taken up by several tumor cells as well as by neutrophils; however, unlike the case with neutrophils, tumor uptake of ZOL results in the stimulation of γδ T cells and an unleashing of their cytotoxic effector functions (69, 122). As shown in Figure 4A, treatment with ZOL in the presence of short-term expanded γδ T cells induced complete lysis of Panc89 cells by activated γδ T cells, which could not be further potentiated by the addition of neutrophils. ZOL further increased the release of granzyme B and IFN-γ by γδ T cells compared to the cultures without ZOL (Figures 4B, 3C). Notably, the presence of ZOL-activated neutrophils additionally enhanced the release of the mediators secreted by γδ T cells in these co-cultures. These results further underline synergistic effects of neutrophils and short-term-activated γδ T cells in the lysis of PDAC cells, and suggest that cytotoxicity of short-term expanded γδ T cells is less susceptible to inhibition by ZOL-activated neutrophils.

## Concluding Remarks

**Figure 5:**
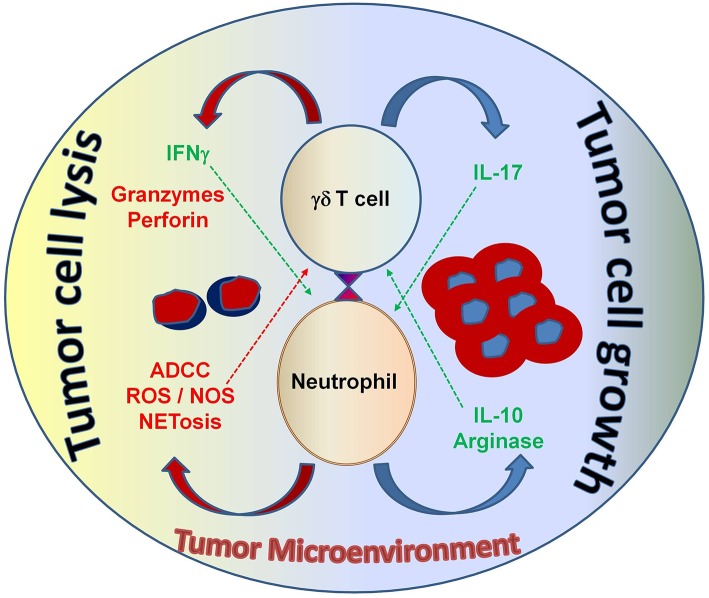
The interaction between neutrophils and γδ T cells can be both pro- and anti-tumorigenic, depending on the tumor microenvironment. Neutrophils can function as myelosuppressive cells and release factors, such as arginase and IL-10 that work to suppress anti-tumor immune effector functions. Alternatively, neutrophils can directly take on anti-tumor effector functions by releasing noxious substances that kill cancer cells. Both tumor-derived factors and accessory immune cells play a role in modulating neutrophil function in the context of malignancy. γδ T cells that secrete IL-17 promote myelosuppressive cells; whereas, γδ T cells that secrete IFN-γ augment anti-tumor effector functions and display potent cytotoxicity, which is important for tumor cell eradication. Understanding this interplay will help develop strategic therapeutic agents that shape the immune environment to elicit strong anti-cancer effector functions from both neutrophils and γδ T cells—with the goal of improving immunotherapy outcomes for patients with cancer. ADCC, antibody-dependent cellular cytotoxicity; ROS, reactive oxygen species; NOS, nitric oxide species; NETosis, the process of cell death induced by the release of chromatin and granular contents into the extracellular space.

Taken together, this study adds to our understanding of how neutrophils can influence γδ T cell-cytotoxicity depending on the situational factors present, such as the activation status of the cells, the cytokine (and chemokine) milieu and the contribution of cytotoxic mediators by other immune cells (e.g., macrophages). Certainly, other important factors that modulate the interaction between neutrophils and γδ T cells in the context of malignancy are also at play—and these include the type or entity of the tumor as well as tumor-derived-signals (e.g., cytokines or damage-associated molecular patterns). The multi-faceted interactions between tumor cells, neutrophils and γδ T cells are graphically summarized in Figure 5. As human γδ T cells infiltrate in many tumors and have attracted much attention for their potential application for cancer immunotherapy (82, 104), partly due to their HLA-independent recognition of antigens, understanding the relationship between neutrophils and γδ T cells is very important. Our study demonstrated that neutrophils can under certain circumstances enhance the killing capacity of short-term expanded γδ T-cell lines by increasing their release of cytotoxic mediators. In on-going studies, we aim to explore whether expansion of γδ T cells by their selective γδ T-cell agonists and enhancing their cytotoxicity by bispecific antibodies can really overcome an immunosuppressive tumor microenvironment, as we have postulated (102, 116). Neutrophils or neutrophil-like cells can infiltrate in tumors, and—given their high heterogeneity and plasticity—are subject to polarization to distinct phenotypes, either promoting tumor development/ progression or killing tumor cells (6, 19, 112). The conditions for neutrophils may be different in the tumor microenvironment compared to peripheral blood. It appears that neutrophils can bind to tumor cells in the bloodstream and transport them to potentially new metastatic sites and conditioning them to support pro-tumorigenic functions (123, 124). In line, an increased number of neutrophils in the blood of cancer patients has been associated with poor clinical outcome (110, 111). Pro-tumorigenic tumor-associated neutrophils (TAN) are described to enhance tumor cell growth and metastasis, support tumor angiogenesis and mediate immunosuppression (19, 112). In contrast, anti-tumorigenic TANs can lyse tumor cells by the release of noxious substances or exert antibody-dependent cellular cytotoxicity (ADCC) by their expression of Fc receptors (19). Neutrophil-mediated ADCC is described by Matlung and colleagues to occur through trogocytosis-related necrosis of tumor cells opsonized by therapeutic monoclonal antibodies like trastuzumab. These observations support the concept that neutrophils can be therapeutically targeted to enhance their cytotoxic activity (19). We recently reported that the bispecific antibody, [(HER2)_2_xCD16], has the potential to enhance cytotoxicity of CD16 (FcRγIII)-expressing γδ T cells as well as NK cells to target HER2-expressing solid tumors (116). The fact that CD16 is also expressed on neutrophils suggests that neutrophils may also be a good target for [(HER2)_2_xCD16] to modulate their anti- tumorigenic properties or to overcome their pro- tumorigenic function.

In conclusion, neutrophil- and neutrophil-like cell subsets play an important role in cancer, and the nature of the function of these cells can influence patient outcomes. Our increasing knowledge of how the behavior of these different neutrophil subsets can be modulated opens the door to exploring new promising strategies that aim to optimize the interaction between neutrophils and γδ T cells to overcome malignancy.

## Materials and Methods

### Tumor Cell Lines

Pancreatic ductal adenocarcinoma cell lines (PDAC) Panc89 and PancTuI were kindly provided by Dr. Christian Röder, Institute for Experimental Cancer Research UKSH/CAU, Kiel. Panc89 cells as well as PancTuI cells were cultured in RPMI 1640 supplemented with 2 mM L-glutamine, 25 mM Hepes, 100 U/mL penicillin, 100 μg/mL streptomycin, 10% FCS (complete medium). For removing adherent tumor cells from flasks, cells were treated with 0.05% trypsin/0.02% EDTA. Mycoplasma negativity was routinely analyzed once per month by RT-PCR and the genotype of PDAC cells was recently confirmed by short tandem repeats analysis.

### Isolation of PBMC, Leukocytes, Neutrophils and Establishment of γδ T-Cell Lines

PMBC as well as leukocytes were isolated from heparinized- or EDTA blood from adult healthy blood donors of the Institute of Immunology. In accordance with the Declaration of Helsinki, all blood donors provided written informed consent, and the study was approved by the relevant institutional review board of Kiel University Medical Faculty (D406/14, D445/18). PBMC were isolated from heparinized blood by Ficoll-Hypaque density gradient centrifugation and leukocytes from EDTA blood of the same donors by lysis of red blood cells using RBC lysing solution (BioLegend; Koblenz, Germany). To separate freshly isolated γδ T cells out of PBMC, a negative selection kit [T cell receptor (TCR) γδ^+^ T Cell Isolation Kit, Miltenyi Biotec, Bergisch Gladbach] was used, according to the manufacturer's instructions. PBMC, leukocytes or negatively isolated γδ T cells were co-cultured with PDAC cells (24 h after their adherence) in medium with 50 U/mL rIL-2 (Novartis, Basel, Switzerland) or stimulated with 2.5 μM zoledronic acid (ZOL, Novartis, Basel, Switzerland) or 1 μg/mL [(HER2)_2_xVγ9] bispecific antibody and rIL-2. Generation and binding capacity of the bsAb [(HER2)_2_xVγ9] is described elsewhere (102). To establish short-term γδ T-cell lines, PBMC were cultured in complete medium, and stimulated with 2.5 μM of ZOL with 50 U/mL rIL-2. 50 U/mL rIL-2 was added every 2 days over a culture period of 14 days. The majority of the γδ T-cell lines had a purity of >97% Vγ9Vδ2 γδ T cells determined by staining the cells with anti-CD3 (clone SK7, BD Biosciences), anti-TCR γδ (clone 11F2, BD Biosciences), anti-Vδ2 (clone Immu389, Beckman Coulter) and anti-Vγ9 [clone 7A5; (125)] mAbs followed by flow cytometry analysis. After two weeks, neutrophils were isolated from EDTA blood samples of the same donors. EDTA blood samples of these donors were treated with RBC lysing solution to eliminate red blood cells. Thereafter, neutrophils were isolated by a negative separation using the EasySep Human Neutrophil Enrichment Kit (#19257; Stem Cell Technologies, Grenoble, France). Isolated cells were routinely stained with anti-CD66b mAb (G0F5, BioLegend, San Diego, CA) and analyzed by flow cytometry. All stained samples were measured on a LRS Fortessa flow cytometer (BD Biosciences) using DIVA 8.0 software.

### Real-Time Cell Analyzer

Cytotoxicity of PBMC, leukocytes, γδ T cells, neutrophils or the combination of the latter two against adherent PDAC cells was measured by a Real Time Cell Analyzer (RTCA, X-Celligence, ACEA, San Diego, CA, USA) in triplicates as described elsewhere (102–104, 116). By using RTCA, the impedance of the cells is monitored *via* electronic sensors located on the bottom of 96-well micro-E-plate every 5 min for up to 24 hrs. To this end, 50 μL medium followed by 50 μL of 5 × 10^3^ adherent PDAC cells/well in complete medium were added to the plates. Impedance of the cells reflects changes in cellular parameters such as cell proliferation, morphological changes (e.g., spreading, adherence) and cell death, and is expressed as an arbitrary unit called cell index (CI). Since the initial adherence in different wells can differ slightly, the CI was normalized to one after having reached the linear growth phase. After 24 h, medium, 2.5 μM ZOL or 1 μg/mL bsAb [(HER2)_2_xVγ9] as indicated were added together with PBMC or leukocytes. Alternatively, previously titrated optimal concentrations of inhibitors were added together with neutrophils and medium or ZOL as indicated in the appropriate figures, 3 h before addition of autologous γδ T-cell lines at the indicated effector/target (E/T) ratio together with 12.5 U/mL rIL-2. A final concentration of 4500 U/mL ROS inhibitor Catalase (Sigma-Aldrich, C3556), 10 nM NOS inhibitor N^G^, N^G^ dimethyl L-arginine (Santa Cruz Biotechnology, Santa Cruz, CA) or 10 μM Peptidyl arginine deiminase (PAD) 4 inhibitor GSK484, which prevents NETosis (Cayman Chemical, Ann Arbor, MI) were added in several experiments. When γδ T-cell lines, neutrophils or both together induced lysis of the PDAC cells, the loss of impedance of PDAC cells is shown as decrease of the normalized CI. PDAC cells were treated with 1 % Triton X-100 (final concentration) as a positive control for killing. All cells were monitored every minute for the indicated time points for analysis of cytotoxicity. The experiments were repeated several times as indicated in the Figure Legends under equal conditions using different donors in independent experiments. By using the RTCA software (version 2.0.0.1301, Copyright © 2004 −2012, ACEA) the raw data files were exported to Microsoft Excel [version 14.0.7128.5000, (32-bit)] for further calculation and described as follows. The mean of Triton-X-100 samples was calculated and defined as 100 % lysis after addition of effector cells. The percentage of lysis of each sample was calculated compared to control sample without effector cells or maximal lysis with Triton-X-100.

### Enzyme-Linked Immunosorbent Assay

Five thousand Panc89 cells were seeded in 96-well flat bottom microtiter plates (Nunc, Wiesbaden, Germany) overnight. After 24 h, medium or a final concentration of 2.5 μM ZOL were added together or not with 250,000 neutrophils/well in complete medium 3 hrs before addition of 125,000 γδ T-cell lines (E/T ratio: 25:1) supplemented with 12.5 U/mL rIL-2 for further 24 hrs. To quantify IFN-γ as well as granzyme B released by γδ T-cell lines co-cultured with PDAC cells in the absence or presence of neutrophils, supernatants were collected after incubation time and stored at −20°C until use. IFN-γ was measured by human IFN-γ DuoSet® ELISA and granzyme B by a human granzyme B sensitive sandwich ELISA (both from R&D System) in duplicates following the procedures outlined by the manufacturer.

### Statistics

Data from at least fifteen donors in independent experiments with three biological replicates were used to test for normal distribution with the Shapiro-Wilk test (Graph pad Prism) followed by a parametrical *t*-test using Microsoft Excel. All statistical tests were two-sided and the level of significance was set at 5%.

## Data Availability

The datasets generated for this study are available on request to the corresponding author.

## Author Contributions

H-HO and DW performed experiments, helped to design the study, and wrote parts of the manuscript. SK contributed to the discussion and wrote parts of the manuscript. DK designed the project and wrote and finalized the manuscript.

### Conflict of Interest Statement

Although not related to this work, SK is the Director of Scientific Innovation at Qu Biologics Inc. The remaining authors declare that the research was conducted in the absence of any commercial or financial relationships that could be construed as a potential conflict of interest.
